# Comprehensive functional splicing analysis of non‐canonical *CNGB3* variants using *in vitro* minigene splice assays

**DOI:** 10.1002/path.6431

**Published:** 2025-04-30

**Authors:** Katharina Rawnsley, Nicole Weisschuh, Susanne Kohl, Peggy Reuter

**Affiliations:** ^1^ Institute for Ophthalmic Research, Centre for Ophthalmology University Hospital Tübingen Tübingen Germany

**Keywords:** achromatopsia, CNGB3, minigene assays, splicing mutation, ACMG/AMP classification, functional assays

## Abstract

Variants in the *CNGB3* gene, encoding the B3‐subunit of the cone photoreceptor cyclic nucleotide gated channel, are a major cause of autosomal recessive achromatopsia, a rare inherited retinal disease. The mutation spectrum of achromatopsia‐associated *CNGB3* variants comprises all types of mutations, including those that are straightforward to evaluate in molecular genetic diagnostics, such as frame‐shifting, nonsense, and canonical splice site variants. Additionally, variants have been identified within splice regions outside the conserved ±1,2 splice site dinucleotides, making their potential impact on disease association challenging to interpret. This poses a major hurdle for clinical interpretation of causality between the patient's genotype and the proposed clinical diagnosis, but also for the inclusion of such patients into clinical trials for gene augmentation therapy, for which only patients with confirmed (likely) pathogenic *CNGB3* variants are eligible. We here performed comprehensive genetic functional analysis of 21 candidate spliceogenic *CNGB3* variants—15 reported and 6 novel variants—by means of *in vitro* minigene splice assays and cDNA analysis, and characterization of spliceogenic events by subcloning, Sanger‐sequencing, and capillary fragment analysis. For 16 variants, an impact on splicing was confirmed, supporting the reclassification of 86% of variants of uncertain significance as likely pathogenic or pathogenic according to the ACMG/AMP guidelines. This reclassification enables the confirmation of patients’ genotypes, both retrospectively and prospectively. © 2025 The Author(s). *The Journal of Pathology* published by John Wiley & Sons Ltd on behalf of The Pathological Society of Great Britain and Ireland.

## Introduction

**Figure 1:**
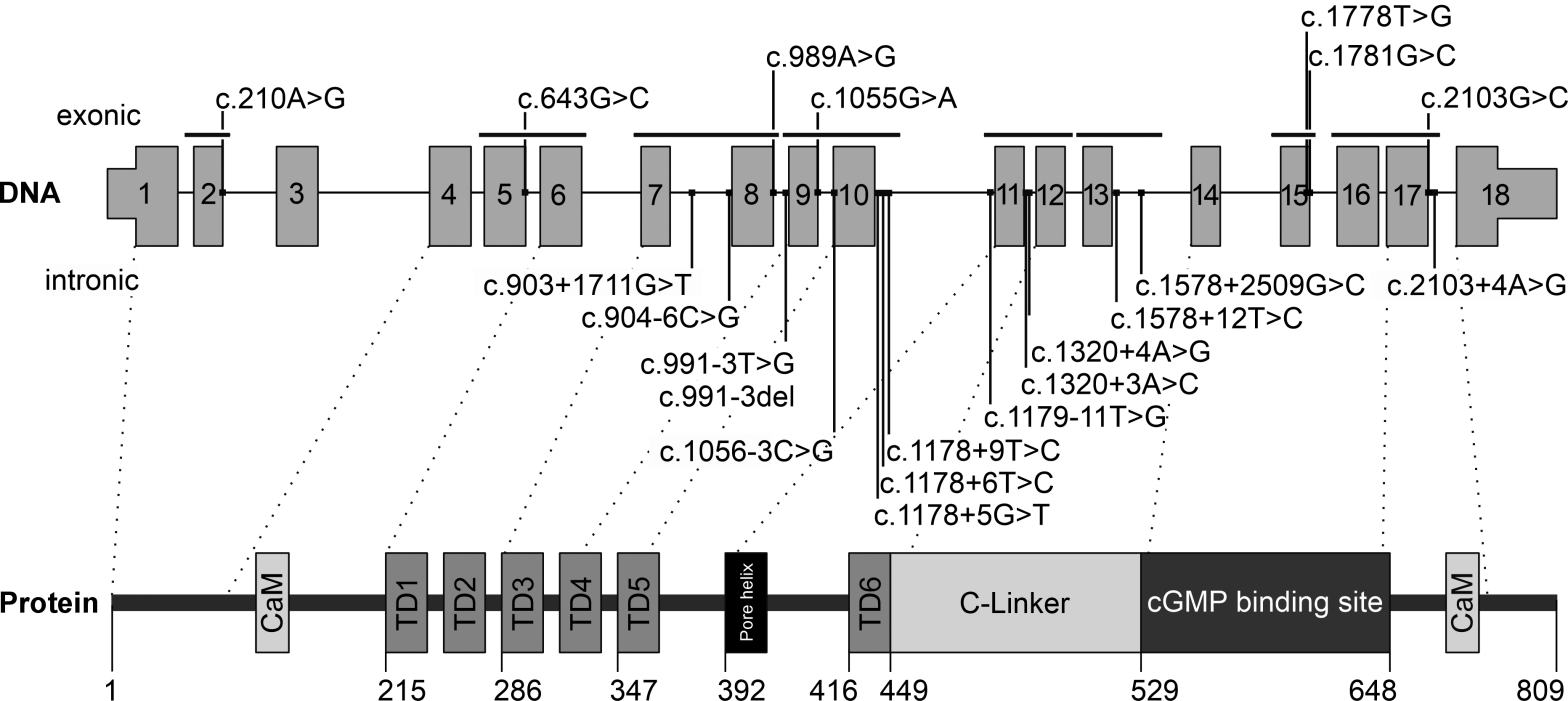
Location of putative splicing variants in *CNGB3*. Upper panel: *CNGB3* gene with 18 coding exons. Exons (gray boxes) and intronic sequence (black horizontal line) are not drawn to scale. Exonic variants are shown above the exons, intronic variants below. Location of splice minigenes are depicted as black lines above the exons. Lower panel: CNGB3 protein with its structural and functional domains including the six transmembrane domains (TD1‐TD6), the pore helix, the C‐linker, the cyclic nucleotide (cGMP) binding domain, and the N‐ and C‐terminal calmodulin (CaM) binding sites. Positions of domains were adapted from [8, 9, 10].

Achromatopsia (ACHM; OMIM 262300; https://www.omim.org/entry/262300) is a rare inherited retinal disease (IRD) characterized by congenital cone dysfunction leading to colorblindness, low visual acuity, disabling sensitivity to light (i.e. photosensitivity), and nystagmus. Pathogenic variants in *CNGB3* (OMIM 605080; https://www.omim.org/entry/605080) are a major cause of ACHM [1, 2, 3, 4, 5]. *CNGB3* encodes for the modulatory B3‐subunit of the cyclic nucleotide‐gated (CNG) channel in cone photoreceptor outer segments [6, 7], a heterotetrameric channel with 3:1 stoichiometry of CNGA3 and CNGB3 subunits [8], respectively (Figure 1). The majority of *CNGB3* variants associated with ACHM are truncating variants, i.e. nonsense, frame‐shifting (i.e. small insertions/deletions/duplications), but also structural variants (i.e. large deletions and duplication) have been found [3]. Missense variants, as well as variants resulting in aberrant splicing, each account for 20% of cases [3, 11], (Human Gene Mutation Database (HGMD®Professional, Cardiff, UK). The deep intronic splicing variant c.1663‐1205G>A represents one of the 10 most recurrent *CNGB3*‐mutant alleles, highlighting the importance of splicing variants in *CNGB3*‐associated ACHM [11].

Splicing variants arise from genetic mutations that disrupt normal pre‐mRNA splicing. These include single nucleotide and small insertion/deletion variants at or near splice sites, or splicing regulatory elements. While it is generally accepted that variants affecting the canonical splice sites are expected to result in aberrant splicing, the impact of non‐canonical variants is elusive. Splicing variants can result in exon skipping, intron retention, or activation of cryptic splice sites, often leading to altered, truncated, or non‐functional proteins. Deep intronic variants, which activate cryptic splice sites resulting in pseudoexon inclusion are increasingly detected through genome sequencing [12, 13, 14, 15, 16, 17].

The disease‐association for non‐canonical putative spliceogenic variants often remains unclear, resulting in their classification as variants of uncertain significance (VUS), according to the guidelines of the American College of Medical Genetics and Genomics/Association for Molecular Pathology (ACMG/AMP). VUS represent a challenge not only in genetic counseling but also in the development of therapies. This is relevant for *CNGB3*, as several gene therapy trials focusing on *CNGB3*‐associated ACHM have been conducted (NCT03001310, NCT03001310, NCT02599922) [18]. For effective patient recruitment and successful study outcome, it is essential to ensure that the disease‐associated variants and genotypes identified in the study participants are truly pathogenic and causative. Functional variant assessment is crucial to achieve this goal.

This study systematically assessed the splicing effects of 21 *CNGB3* variants outside canonical splice sites using *in vitro* minigene assays in HEK293T cells, cDNA analysis, and subcloned reverse‐transcription polymerase chain reaction (RT‐PCR) fragment sequencing, complemented by capillary fragment analysis for selected variants.

## Methods

### Ethics approval

The study was approved by the Ethics Board of the Medical Faculty, University of Tübingen (project no. no. 116/2015BO2, last approval 09 February 2022). Written informed consent of patients or legal guardians was obtained for all patients.

### Nomenclature

Variant nomenclature follows Human Genome Variation Society recommendations and is based on GenBank accession numbers NG_016980.1, NM_019098.5 and NP_061971.3.

### Variant acquisition

The *CNGB3* variant sample set was compiled from our in‐house RetDis patient database including our comprehensive ACHM cohort (>1,000 patients of ~900 independent families) [1, 3], by literature search and database review [i.e. HGMD®, ClinVar (https://www.ncbi.nlm.nih.gov/clinvar/, 25 November 2022), Leiden Open Variation Database (LOVD, https://www.lovd.nl/, 25 November 2022)]. For local cases, written consent was obtained, and the study was approved by the local Ethics Board (project no. 116/2015BO2). Novel variants were submitted to the LOVD (26 September 2024). Only single‐nucleotide variants with an association with ACHM or IRD were considered. The selection of variants included rare exonic and intronic variants within the donor/acceptor splice region (donor: −3 to +6; acceptor: −20 to +3) [19], as well as deep intronic variants for which an effect on splicing was predicted by SpliceAI. Variants affecting the invariable ±1,2 dinucleotides were excluded. However, variant c.1778T>G (fourth to last nucleotide of exon 15) was included due to a predicted splicing effect by SpliceAI. Variants c.1578+12T>C and c.1178+9T>C were also included, as they were classified as VUS at the time of selection, prior to the recommendations by Walker *et al* [19].

### Variant classification

Variant classification was performed using the Franklin classification tool (https://franklin.genoox.com), followed by manual revision based on the most recent recommendation for classification of splicing variants [19, 20]. Population frequency data were retrieved from gnomADv4.0.0 (https://gnomad.broadinstitute.org/). A GroupMax filtering allele frequency above 0.0042 was applied as a threshold for the BS1 (Benign Strong 1) criterion. This threshold was calculated using the Allele Frequency App from CardioDB (https://cardiodb.org/allelefrequencyapp/) [21], based on an ACHM prevalence of 1:30,000, a genetic heterogeneity of 0.78, a penetrance of 0.9, and an allelic heterogeneity of 0.78. The PM2 (Pathogenic Moderate 2) criterion was applied for the maximal allele frequency in subpopulations with a threshold below 0.0027.

### *In silico* splice predictions

*In silico* splice predictions were performed using SpliceAI (https://spliceailookup.broadinstitute.org/), TraP Score [22], MaxEntScan [23], and NNSplice [24]. With the latter tools, splice site strength for wildtype and the mutant sequence was determined independently, and the change in percentage was calculated as described by Jang *et al* [25]. In order to facilitate comparison of the degree of splice change, the absolute value of the score was considered for MaxEntScan, which also returns negative values. Cutoff thresholds were defined as ≥0.2 for SpliceAI, ≥0.289 for non‐coding, and ≥0.416 for coding variants using the TraP Score, and ≥30% for MaxEntScan and NNSplice [19, 26].

### Minigene assays

Minigene assays were generated using the pSPL3 exon trapping plasmid vector, as previously described [27, 28]. An optimized version of the pSPL3 was generated by inverse PCR using primers 5’‐ATTGAAGAATCGCAAAACCAGC‐3’ and 5’‐GAGTGGTGGTTGCTTCCTTC‐3’, with PfuUltra High‐Fidelity DNA Polymerase (Agilent, Santa Clara, CA, USA). Part of the HIV intron was deleted from pSPL3 to reduce the number of cryptic splice sites and to prevent recognition of an occasionally occurring pseudoexon [4, 29]. Inserts included one or two exons of *CNGB3* with variable lengths (>200 bp) of flanking 5’ and 3’ intronic sequences (Figure 1, supplementary material, Table S1). *CNGB3* fragments of interest were PCR amplified from human genomic DNA of a healthy subject using a proofreading DNA polymerase and cloned into the pSPL3 minigene plasmid. Wildtype minigene constructs were verified by Sanger sequencing and cDNA analysis to check for correctly spliced mRNA. Twenty‐one variants of interest were introduced into the wildtype constructs by site‐directed *in vitro* mutagenesis, applying the QuikChange® Site‐Directed Mutagenesis Kit (Stratagene, La Jolla, CA, USA). Primers were designed using the PrimerX online tool (https://www.bioinformatics.org/primerx/index.htm). Mutant minigenes were also verified by Sanger sequencing of all exons as well as flanking intronic sequences applying the SupreDye™ v1.1 Cycle Sequencing Kit (AdvancedSeq LLC, Livermore, CA, USA).

For comparative minigene assays, HEK293T/17 cells (ATCC® CRL‐11268™) (https://www.atcc.org, Manassas, VA, USA) at 80% confluency were transfected in 24‐well plates using 2 μg of mutant or wildtype minigenes plasmid DNA and 4 μl lipofectamine™ 2000 (Thermo Fisher Scientific, Dreieich, Germany) following the manufacturer's protocols. At least two independent transfections were performed. Total RNA was extracted using the PeqGold Total RNA Kit (VWR, Darmstadt, Germany) 24 h post‐transfection, and 1 μg RNA was reverse‐transcribed using the FAST cDNA Synthesis Kit (7BioScience GmbH, Neuenburg am Rhein, Germany) and pSPL3 transcript‐specific primer 5’‐ATCTCAGTGGTATTTGTGAGC‐3’. Splice products were PCR‐amplified using forward 5’‐TGGACAACCTCAAAGGCACC‐3’ and reverse primer 5’‐AGTGAATTGGTCGAATGGATC‐3’ applying 2 μl cDNA synthesis reaction and 35 cycles. RT‐PCR products were separated and visualized on ethidium bromide‐stained agarose gels, and fragments were either directly sequenced or subcloned using the CloneJET PCR Cloning Kit (Thermo Fisher Scientific) following the manufacturer's protocol. At least eight clones per fragment were Sanger‐sequenced.

### Capillary fragment analysis

Capillary fragment analysis was performed on selected variants to quantify aberrantly spliced RT‐PCR products of mutant minigenes, in comparison to the corresponding wildtype minigenes. Splice products were PCR‐amplified using a FAM‐fluorophore‐coupled version of the reverse primer, as described above (MERCK, KGaA, Darmstadt, Germany). RT‐PCR products were run on the ABI PRISM 3130xl Genetic Analyzer (Thermo Fisher Scientific) using MapMarker ROX 1000 (BioVentures, Murfreesboro, TN, USA) as a size standard. Data were analyzed using GeneMapper® Software 5 (Applied Biosystems™, Foster City, CA, USA; Thermo Fisher Scientific).

### Evaluation of RNA‐seq data

Three bulk RNA‐seq datasets (SRR5225779, SRR5225767, SRR5225763; NCBI BioProject PRJNA369687) [30] from human *postmortem* macular retina of healthy donors were analyzed. Sample quality was assessed using FastQC (http://www.bioinformatics.babraham.ac.uk/projects/fastqc/). Adapter trimming was performed using Trim Galore! (https://github.com/FelixKrueger/TrimGalore), and reads were mapped to the GRCh38/hg38 reference genome using HISAT2 [31]. Data visualization and Sashimi plot generation were performed using the Integrative Genomics Viewer (IGV 2.16.2) [32, 33].

## Results

Twenty‐one *CNGB3* variants were assessed for their potential to affect splicing of *CNGB3* (Table 1, Figure 1, and supplementary material, Table S2). Fifteen single‐nucleotide variants were selected from public databases (HGMD®, LOVD, ClinVar), and six variants were found in our local ACHM database (full genotypes provided in supplementary material, Table S3). Of the 21 variants studied, seven variants were located within the exonic sequence, and 10 within the intronic sequence of the consensus donor or acceptor site motifs. Two variants (c.1178+9T>C and c.1578+12T>C) were situated outside the consensus sequence, and two others (c.903+1711G>T and c.1578+2509G>C) were found deep within the intronic sequence.

**Table 1:** *CNGB3* variants analyzed in this study and corresponding *in silico* splice prediction, outcome of minigene assay, and predicted consequence on protein structure

Variant transcript^‡^	Location/Minigene	*In silico* splice prediction				Minigene splice assay results^£^	Predicted effect on protein^$^
		Splice AI ∆score°	TraP score^†^	Max Ent Scan^#^ [%]	NN Splice^¥^ [%]		
c.210A>G	Exon 2 dsrMG Ex2	**Donor loss** **0.65 (−1 bp)** Acceptor loss 0.57 (80 bp)	**0.97**	**47.2**	**−100.0**	**Major transcript: Skipping of exon 2 (82 bp)**	p.(E44Tfs*12) 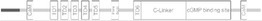
						Minor transcript: Spliced correctly	Wildtype 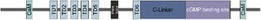
c.643G>C	Exon 5 dsr MG Ex5–6	**Donor loss** **0.54 (0 bp)** Acceptor loss 0.55 (149 bp)	**0.90**	**119.4**	**−100.0**	**Skipping of exon 5 (150 bp)**	p.(A165_T214del) 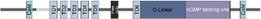
c.903+1711G>T	Intron 7 div MG Ex7–8	**Acceptor gain 0.41 (−18 bp)** Donor gain 0.26 (**−**49 bp)	0.04	10.2	No splice site detected	**Major transcript: Pseudoexon (32 bp) inclusion**	p.(L302Sfs*17) 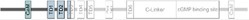
						Minor transcript: Pseudoexon (32 bp) inclusion and skipping of exon 8 (87 bp)	p.(L302Sfs*22) 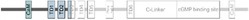
						Minor transcript: Spliced correctly	Wildtype 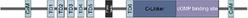
						Minor transcript: Skipping of exon 8 (87 bp)	n.a.*
						Minor transcript: Skipping of exon 7 (51 bp) and exon 8 (87 bp)	n.a.*
c.904‐6C>G	Intron 7 asr MG Ex7–8	**Acceptor loss 0.48 (−6 bp)** Donor loss 0.31 (**−**92 bp)	**0.51**	14.4	No splice site detected	**Skipping of exon 8 (87 bp)**	p.(L302_K330del) 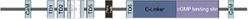
c.989A>G	Exon 8 dsr MG Ex7–8	**Donor loss** **0.68 (−1 bp)** Acceptor loss 0.50 (85 bp)	**0.98**	12.9	**−**1.0	**Skipping of exon 8 (87 bp)**	p.(L302_K330del) 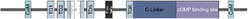
c.991‐3T>G	Intron 8 asr MG Ex7–8	**Acceptor loss 0.95 (−3 bp)** Acceptor Gain 0.99 (**−**1 bp)	**0.58**	**224.2**	5.3	**Newly formed splice acceptor site used (inclusion of 2 bp of intron 8)**	p.(Y331Sfs*12) 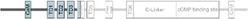
c.991‐3del	Intron 8 asr MG Ex7–8	**Acceptor loss 0.50 (−2 bp)** Acceptor loss 0.50 (85 bp)	n/a	**252.9**	**−100.0**	**Skipping of exon 9 (65 bp)**	p.(Y331Sfs*15) 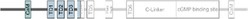
c.1055G>A	Exon 9 dsr MG Ex9–10	**Donor loss 0.89 (0 bp)** Acceptor loss 0.95 (64 bp)	**0.99**	**72.1**	**−100.0**	**Skipping of exon 9 (65 bp)**	p.(Y331Sfs*15) 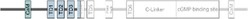
c.1056‐3C>G	Intron 9 asr MG Ex9–10	**Acceptor loss 0.97 (−3 bp)** Donor loss 0.96 (**−**125 bp)	**0.64**	**174.0**	**−100.0**	**Skipping of exon 10 (123 bp)**	p.(V353_E393del) 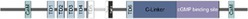
c.1178+5G>T	Intron 10 dsr MG Ex9–10	**Donor loss 0.98 (5 bp)** Acceptor loss 0.93 (127 bp)	**0.92**	**77.6**	**−100.0**	**Major transcript: Skipping of exon 10 (123 bp)**	p.(V353_E393del) 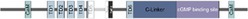
						**Major transcript: Skipping of exon 10 (123 bp)**	p.(V353_E393del) 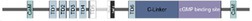
						Minor transcript: Skipping of exon 9 (65 bp) and Exon 10 (123 bp)	p.(Y331Vfs*26) 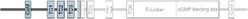
c.1178+6T>C	Intron 10 dsr MG Ex9–10	**Donor loss** **0.73 (6 bp)** Acceptor loss 0.60 (128 bp)	**0.92**	**31.9**	**−**9.1	**Major transcript: Spliced correctly**	Wildtype 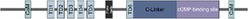
						Minor transcript: Skipping of exon 9 (65 bp) and exon 10 (123 bp)	p.(V353_E393del) 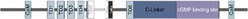
						Minor transcript: Skipping of exon 10 (123 bp)	p.(Y331Vfs*26) 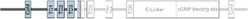
c.1178+9T>C	Intron 10 dsr MG Ex9–10	Donor loss 0.00 (9 bp)	0	n/a	0.0	**Spliced correctly**	Wildtype 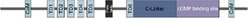
c.1179‐1111T>G	Intron 10 asr MG Ex9–10	**Acceptor loss 0.68 (−11 bp)** Donor loss 0.69 (**−**152 bp)	**0.36**	14.3	**−**2.0	**Skipping of exon 11 (142 bp)**	p.(Y394*) 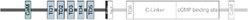
c.1320+3A>C	Intron 11 dsr MG Ex11–12	**Donor loss** **0.92 (3 bp)** Acceptor loss 0.79 (144 bp)	**0.93**	27.5	**−33.3**	**Skipping of exon 11 (142 bp)**	p.(Y394*) 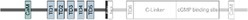
c.1320+4A>G	Intron 11 dsr MG Ex11–12	**Donor loss 0.69 (4 bp)** Acceptor loss 0.75 (145 bp)	**0.92**	0.0	**−**9.1	**Skipping of exon 11 (142 bp)**	p.(Y394*) 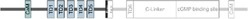
c.1578+12T>C	Intron 13 dsr MG Ex13	Donor loss 0.02 (12 bp)	0.01	n/a	no splice site detected	**Major transcript: Spliced correctly**	Wildtype 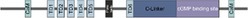
						**Minor transcript: Skipping of exon 13 (98 bp)**	n.a.*
c.1578+2509G>C	Intron 13 div MG Ex13	**Acceptor gain 0.91 (−3 bp)** Donor gain 0.75 (**−**100 bp)	0.02	**575.6**	**100.0**	**Pseudoexon (98 bp) inclusion**	p.(G527Dfs*24) 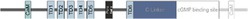
c.1778T>G	Exon 15 dsr MG Ex15	**Donor loss 0.24 (−3 bp)** Acceptor loss 0.79 (144 bp)	**0.58**	n/a	20.59	**Major transcript: Spliced correctly**	p.(I593S) 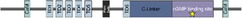
						Minor transcript: Cryptic exonic donor used (deletion of last 52 bp of exon 15)	p.(G577Afs*3) 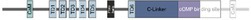
						Minor transcript: Skipping of exon 15 (119 bp)	p.(G555Pfs*41) 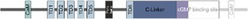
c.1781G>C	Exon 15 dsr MG Ex15	**Donor loss 0.72 (0 bp)** Acceptor loss 0.78 (118 bp)	**0.87**	**62.2**	**−100.0**	**Major transcript: Skipping of exon 15 (119 bp)**	p.(G555Pfs*41) 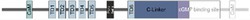
						Minor transcript: Cryptic exonic donor use (deletion of last 52 bp of Exon 15)	p.(G577Afs*3) 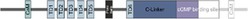
c.2103G>C	Exon 17 dsr MG Ex16–17	**Donor loss 0.97 (0 bp**) Donor gain 0.68 (−51 bp)	**0.84**	**79.9**	**−100.0**	**Major transcript: Cryptic intronic donor used (first 51 bp of intron 17 retained)**	p.(Q701_Q809delinsHVIRWSEWTAVMEKAPD*) 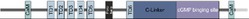
						Minor transcript: Cryptic intronic donor used (first 8 bp of intron 17 retained)	p.(Q701Hfs*131) 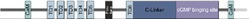
						Minor transcript: Skipping of exon 16 (147 bp) and exon 17 (175 bp)	n.a.*
c.2103+4A>G	Intron 17 dsr MG Ex16–17	Donor loss 0.16 (4 bp) Donor gain 0.37 (**−**47 bp)	**0.92**	**31.1**	**−**18.1	**Major transcript: Spliced correctly**	Wildtype 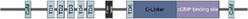
						Minor transcript: Cryptic intronic donor used (first 51 bp of intron 17 retained)	p.(K702_Q809delinsVVRWSEWTAVMEKAPD*) 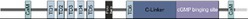
						Minor transcript: Skipping of exon 16 (147 bp) and exon 17 (175 bp)	n.a.*

*Note*: Variant transcript^‡^: NCBI Reference Sequence: NM_019098.5 (https://www.ncbi.nlm.nih.gov/nuccore/NM_019098.5/). Splice AI ∆score°: scores in bold indicate a change of ∆score ≥0.2. Positions given are relative to the variant, with negative values being located to the left of the variant in genomic coordinates on the forward strand. Additional predicted changes affecting neighboring splice site are shown in parentheses. TraP score^†^: cutoff for TraP score: non‐coding 0.289 & coding 0.416, values above cutoff are depicted in bold [26]. Max Ent Scan^#^: Absolute change in strength relative to wildtype splice site; % in bold represent ≥30% change. NN Splice^¥^: Change in strength relative to wildtype splice site, with % in bold representing ≥ ± 30% change. Minigene splice assay results^£^: Major and minor refer to the relative strength of transcripts observed on agarose gels and/or the relative ratios after subcloning or capillary fragment analysis. Predicted effect on protein^$^: Predicted effect of aberrant splicing on protein.

Abbreviations: asr, acceptor splice region; bp, base pairs; div, deep intronic variant; dsr, donor splice region; MG, minigene construct; n.a., not analyzed; n/a, not applicable.

* These RT‐PCR products were also detected as minor transcripts in the corresponding wildtype minigene and are therefore not classified as relevant aberrant splice products.

*In silico* splice prediction using four different tools (i.e. SpliceAI, TraP Score, MaxEntScan, and NNSplice) concordantly suggested an effect on splicing for eight, and no effect for two variants (c.1178+9T>C and c.1578+12T>C) (Table 1). Non‐uniform predictions were obtained for the remaining 11 variants.

**Figure 2:**
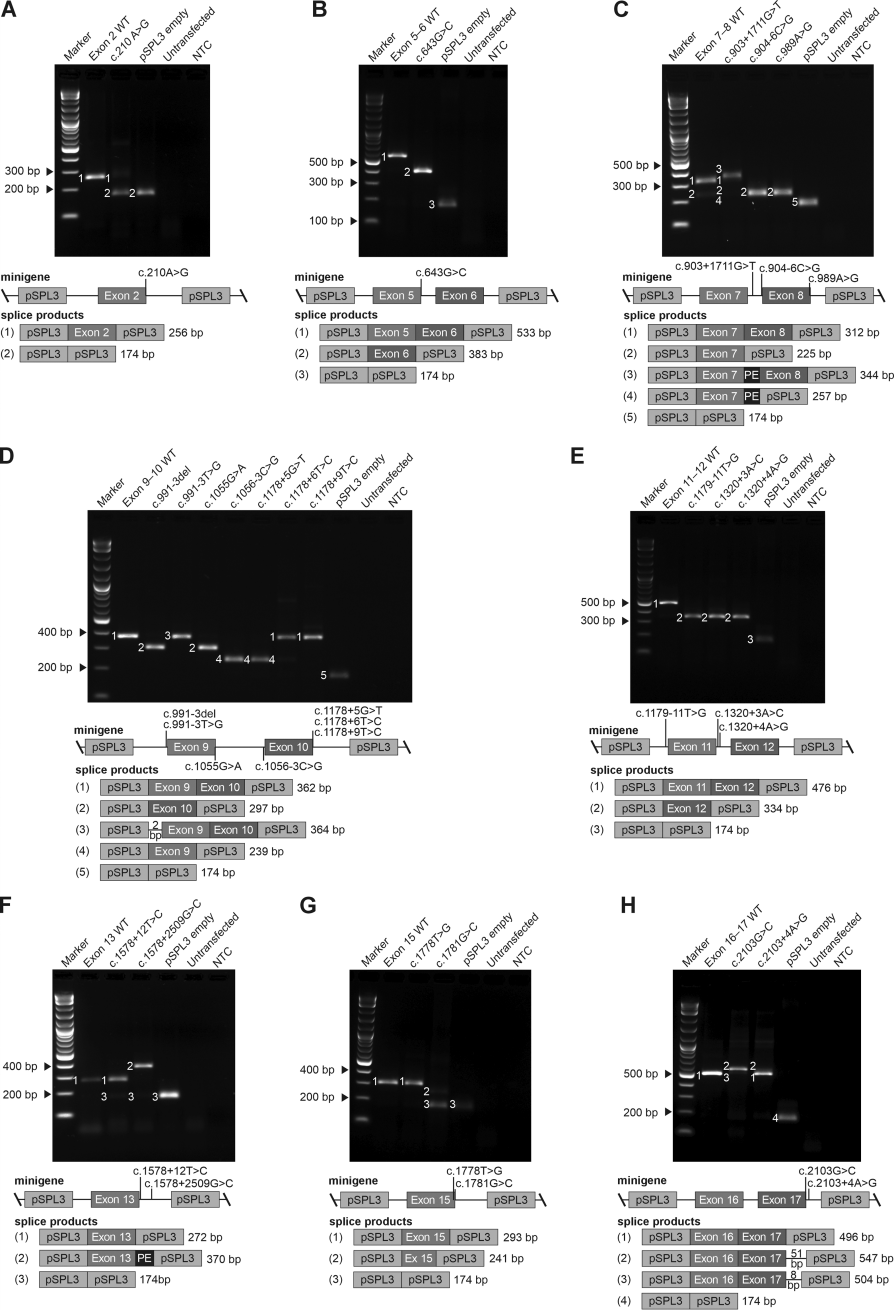
Splicing outcome for the *CNGB3* variants tested by minigenes in (A) *CNGB3* exon 2, (B) exons 5–6, exons (C) 7–8, (D) exons 9–10, (E) exons 11–12, (F) exon 13, (G) exon 15, and (H) exons 16–17. For all assays and composite images: Top: RT‐PCR products separated and visualized using agarose gel electrophoresis and staining with ethidium bromide. From left to right: Size standard (1 kb plus DNA ladder); RT‐PCR product from the respective wildtype (WT) minigene; RT‐PCR product(s) from the respective mutant minigene(s); RT‐PCR product from the empty pSPL3 vector, RT‐PCR from non‐transfected HEK293T cells, and no template control (NTC). Observed transcripts are labeled with numbers, which correspond to the splice products in the lower panel. Note that not all transcripts identified by subcloning/capillary fragment analysis are visible on the agarose gel, and, conversely, not all bands on the gel could be captured by subcloning. Middle: Respective minigene construct including pSPL3 vector specific exons (gray boxes), *CNGB3* exons and location of variants tested in each assay. Bottom: Schematic representation of observed transcripts in the splice assays, including the size of the RT‐PCR product. Detailed information on the retained intronic sequences including size in bps are given in the supplementary material, Figure S1 (wildtype constructs) and Figure S2 (variant constructs). PE: pseudoexon.

Eight different wildtype minigenes were generated and evaluated in HEK293T cells (Figure 1) using the empty pSPL3 and non‐transfected HEK293T cells as controls. For all wildtype minigenes, RT‐PCR products representing correctly spliced transcripts could be visualized by agarose gel electrophoresis (Figure 2), and confirmed by subcloning and/or Sanger‐sequencing (supplementary material Figure S1 and Table S4). In addition, (very) low levels of alternatively spliced transcripts (*CNGB3* exon skipping) were detected in 4/8 minigenes on agarose gel or following subcloning (Figure 2, supplementary material Figure S1 and Table S4). Alternative splicing of *CNGB3* exons has not been previously reported and was also not observed in bulk RNA‐seq data of *postmortem* human macular retina (supplementary material, Figure S2). Therefore, skipping of exons (from now on referred to as ΔEx) in the wildtype minigene transcripts is likely due to the experimental setup. In the minigene Ex7–8, a small fraction of transcripts containing a 32‐bp pseudoexon, located between exons 7 and 8, was detected by subcloning and capillary fragment analysis (supplementary material, Figure S4 and Table S4). In one of three human macular retinal RNA‐seq samples assessed, recognition of this pseudoexon within intron 7 was also observed in few reads, suggesting that this aberrant splicing event occurs not only in HEK293T but also in retinal cells (supplementary material, Figure S2). Since for each wildtype minigene examined, the strongest RT‐PCR product (as judged from the relative strength of the fragments observed on the agarose gel or capillary fragment analysis) corresponded to the correctly spliced transcript, all eight wildtype minigenes were considered suitable for assessing the 21 potential spliceogenic *CNGB3* variants.

Of the 21 mutant minigenes tested, no aberrant splicing was observed for variants c.1178+9T>C and c.1578+12T>C (Figure 2D,F). A small proportion of Δ(Ex13) was detected for the c.1578+12T>C minigene by agarose gel electrophoresis and subcloning, similar to the wildtype Ex13 minigene. Therefore, Δ(Ex13) was not considered an indicator of aberrant splicing induced by c.1578+12T>C (supplementary material, Table S4).

Aberrant splicing was detected for 19 mutant minigenes (Figure 1, Table 1, and supplementary material, Figure S3). For 16 variants, aberrant splicing was detected, whereby for 11/16 of the mutant minigenes only one aberrant transcript and for 5/16 variants several aberrant transcripts were observed. Of these 16 variants, six were exonic (one synonymous, five predicted missense). Therefore, the synonymous variant can be considered as a spliceogenic variant and the predicted missense variants likely exert their pathogenic effect by inducing aberrant splicing rather than through amino acid substitution.

The most commonly observed aberrant splicing event was exon skipping. The second most common mechanism was use of cryptic exonic or intronic splice acceptor (e.g. c.991‐3T>G) or donor sites (e.g. c.2103G>C, c.2103+4A>G, 1778T>G, c.1781G>C) resulting in partial intron retention or exon deletion. For both deep intronic variants (c.903+1711G>T and c.1578+2509G>C), pseudoexon inclusion was demonstrated. Yet while this was the solely induced event for c.1578+2509G>C, the aberrant splicing profile for variant c.903+1711G>T was more complex: minor transcripts with pseudoexon inclusion and Δ(Ex8), Δ(Ex8) alone, or Δ(Ex7/8), but also correctly spliced transcripts were observed following subcloning. The relevance of these latter minor transcripts remains elusive, as these were also observed for the Ex7–8 wildtype minigene (supplementary material, Figures S4–S6). Therefore, capillary fragment analysis was performed to quantify the ratio between the different observed transcripts in the wildtype and the variant minigene constructs (supplementary material, Figures S4–S6). It is important to note, that while we decided to perform this extended analysis, we do not propose that the relative expression ratios necessarily reflect the ratios that would be observed in the native situation, i.e. in the cone photoreceptors.

For wildtype minigene Ex7–8, the correctly spliced transcript including exons 7 and 8 was the predominant transcript (61.4 ± 2.5%), and the second most prominent transcript showed Δ(Ex8) (37.3 ± 4.3%) (supplementary material, Figure S4A). Δ(Ex7/8) was detected in 0.2 ± 0.3% of transcripts. The inclusion of a 32‐bp pseudoexon between exons 7 and 8 was also detected at a low level (1.1 ± 1.6%) in the wildtype minigene Ex7–8 transcripts. For the mutant minigene, the fraction of transcript including the pseudoexon increased to 56.4 ± 8.5%, indicating enhanced pseudoexon recognition due to the variant c.903+1711G>T (supplementary material, Figure S4B). In line with this, the amount of correctly spliced transcripts was reduced for the mutant minigene (25.9 ± 6.7% exons 7 and 8 inclusion).

Of the 19 mutant minigenes showing aberrant splicing, three variants (c.1178+6T>C, c.1778T>G, c.2103+4A>G) gave rise to primarily correctly spliced and only small amounts of aberrant transcripts. For c.1178+6T>C, aberrant splicing was evident through agarose gel electrophoresis, while aberrant transcripts for c.1778T>G and c.2103+4A>G were detected only after subcloning. Capillary fragment electrophoresis confirmed that these aberrant transcripts were variant‐specific and absent in wildtype controls. For c.1778T>G, 0.6 ± 0.1% of transcripts lacked 52 bp of exon 15 due to the use of a cryptic splice site. A slight increase in Δ(Ex15) transcripts was observed (9.1 ± 0.9% for c.1778T>G versus 5.9 ± 0.4% for wildtype), while 90.2 ± 0.8% were correctly spliced, similar to the wildtype (94.1 ± 0.4%). For the c.2103+4A>G minigene, an increase (22.3 ± 3.0%) in Δ(Ex16/17) transcripts (2.5 ± 3.5% for wildtype) and minor amounts (4.0 ± 0.5%) of transcripts with intron retention were detected—the latter was absent in the wildtype. Since the pathogenicity of these low levels of aberrant transcripts remains unclear, these three variants are classified as hypomorphic.

For most transcripts, the aberrant splicing was predicted to result in either frame‐shift and premature termination codons (PTC) or direct PTC. These transcripts, if expressed and translated, would result in a truncated CNGB3 protein, with either loss of all or several structural and functional relevant domains (Table 1—Predicted effect on protein). However, these aberrant transcripts are likely to be degraded by nonsense‐mediated mRNA decay (NMD). In contrast, six variants (c.643G>C, c.904‐6C>G and c.989A>G, c.1056‐3C>G, c.1178+5G>T, and c.1178+6T>C) induced in‐frame deletions, i.e. Δ(Ex5), Δ(Ex8), or Δ(Ex10), respectively. These deletions result in the loss of either 50 amino acid (aa) residues between the N‐terminal CaM binding site and transmembrane domain (TD) 1 (p.A165_T214del), 29 aa residues spanning TD3 to TD4 (p.L302_K330del), or 41 aa residues within TD5 and the pore (p.V353_E393del), respectively (Table 1).

Finally, the results of the functional *in vitro* minigene assay were correlated with predictions from the four different *in silico* tools (Table 1). The *in vitro* minigene assays revealed that variants c.1178+9T>C and c.1578+12T>C did not affect splicing. These findings correlate well with the predictions of SpliceAI, TraP score, and for the latter variant, NNSplice. MaxEntScan was not applicable for these sites. For the 19 variants that impaired splicing of *CNGB3*, SpliceAI and TraP score were the most reliable tools. SpliceAI failed only for the hypomorphic variant c.2103+4A>G, while the TraP score was found to be not suitable for the prediction of deep intronic variants. Overall, SpliceAI had an accuracy of 95%, followed by TraP Score at 90%. MaxEntScan and NNSplice proved to be less reliable, with accuracies of 67% and 61%, respectively. In addition, SpliceAI performed well with predicting aberrant splice events, such as exon skipping and enhanced use of cryptic intronic donors. For the two deep intronic variants, SpliceAI not only correctly predicted the new acceptor splice sites formed/strengthened by the variants, but also identified the correct downstream donor, leading to the 32 bp and 98 bp pseudoexons observed for the variants c.903+1711G>T and c.1578+2509G>C, respectively.

**Figure 3:**
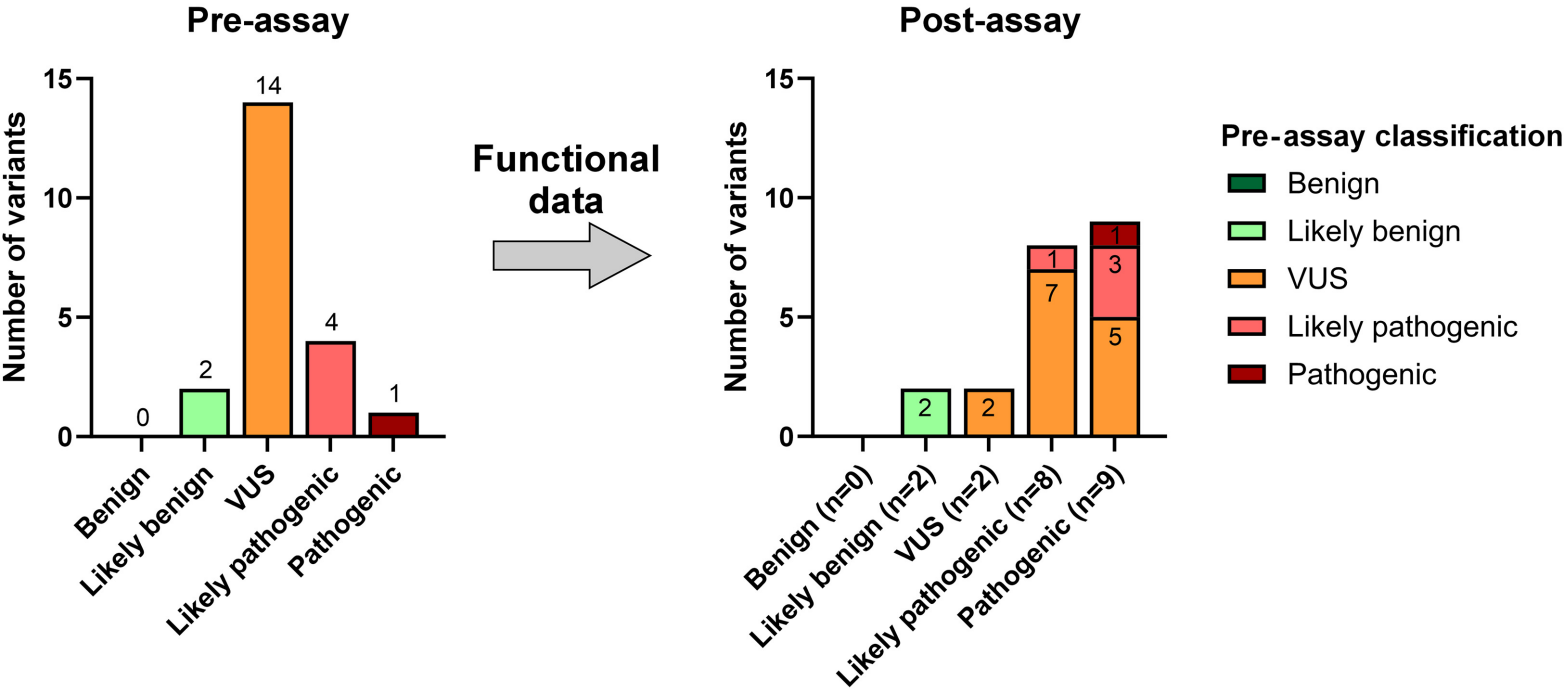
Influence of functional data on the ACMG/AMP‐based variant classification of 21 analyzed *CNGB3* putative splicing variants. Pre‐assay ACMG/AMP classification of the 21 *CNGB3* variants resulted in two likely benign, 14 variants of uncertain significance (VUS), four likely pathogenic, and one pathogenic variant (left). Incorporating the results of functional analysis using minigene assays allowed confirmation and reclassification in the post‐assay ACMG/AMP classification (right). Two likely benign variants were confirmed, as was one a pathogenic variant. Seven VUS were reclassified to likely pathogenic, and five to pathogenic. Three likely pathogenic variants could be reclassified as pathogenic. Only two variants remained as VUS post‐assay as well as one likely pathogenic variant.

Prior to the functional assay, the ACMG/AMP classification categorized the variants as follows: two (9.5%) were classified as benign, 14 (67%) as VUS, four (19%) as likely pathogenic, and one (5%) as pathogenic (Figure 3, supplementary material, Table S2). For the 16 spliceogenic variants, the *in vitro* minigene assay results were applied to the ACMG/AMP classification using the PVS1 (Pathogenic Very Strong 1) criterion, at varying strengths [19, 20]. The pre‐ and postassay classifications are provided in supplementary material, Table S2. For the three hypomorphic variants (c.1178+6T>C, c.1778T>G, c.2103+4A>G), neither the PVS1 nor the BP4 (Benign Prediction 4) criterion was applied. Therefore, the classification of the two VUS (c.1178+6T>C and c.2103+4A>G) and the one likely pathogenic variant c.1778T>G remained unchanged. Adding the functional evaluation evidence resulted in the upgrade of seven (33%) VUS to likely pathogenic (c.210A>G, c.643G>C, c.903+1711G>T, c.904‐6C>G, c.989A>G, c.1056‐3C>G, c.2103G>C) and five (24%) VUS to pathogenic (c.991‐3del, c.1055G>A, c.1179‐11T>G, c.1320+4A>G, c.1578+2509G>C). Three (14%) likely pathogenic variants (c.1178+5G>T, c.1320+3A>C, c.1781G>C) were upgraded to pathogenic. The classification of two variants (c.1178+9T>C, c.1578+12T>C) as likely benign was confirmed, along with the classification of one pathogenic variant (c.991‐3T>G). Initial classification had categorized 14 (67%) variants as VUS, and the functional assay enabled reclassification of 86% of these, leaving only two (9.5%) in this inconclusive category, which is considered one of the most important outcomes of the study.

## Discussion

Variants in *CNGB3* cause ACHM, and several clinical trials have used gene augmentation therapy to try to treat this disease (NCT03001310, NCT03001310, NCT02599922) [18]. Ideally, only patients with biallelic (likely) pathogenic variants should be included in such trials. Yet for the classification of missense, late nonsense, and putative splicing variants, this relies on the functional confirmation of the pathogenic effect of such a variant. This is challenging, and depends on developing adequate functional studies, which is often limited to *in vitro* assays, as cells and tissue expressing the gene of interest may not be accessible.

Our study focused on evaluating the splicing effects of 21 single‐nucleotide variants identified within the *CNGB3* gene in association with IRD. *In silico* predictions using multiple tools indicated spliceogenic potential for most variants. However, the accuracy and concordance of these predictions were variable, highlighting and necessitating experimental validation through minigene assays. When comparing the results of the applied *in silico* prediction tools, the SpliceAI algorithm was the most informative tool, with an accuracy of 95%.

Minigene assays have been designated as useful tools for assessing the effect of variants on splicing by the ClinGen SVI Splicing Subgroup [19]. Especially for genes like *CNGB3*, where patient‐derived tissue samples expressing the gene of interest (i.e. retina and cone photoreceptors) are impossible to obtain, it is a versatile and cost‐effective approach. In IRD, numerous gene‐specific studies have been conducted using *in vitro* minigene splice assays for the validation of splicing variants (e.g. *ABCA4*, *CNGA3*, *USH2A*, *PRPF31*, *IFT140*, and *CRB1*) [17, 28, 34, 35, 36]. In the present study, eight wildtype minigene constructs were generated, each encompassing specific exons and their flanking intronic regions of *CNGB3*. Analysis of these constructs confirmed the presence of correctly spliced wildtype‐like transcripts, as expected, but also revealed minor aberrant transcripts in these. The latter resulted from alternative splicing of the *CNGB3* exons in the HEK293T cell culture system. Since alternative splice isoforms for *CNGB3* have previously not been described, and were also not detected in human macular retinal RNA‐seq data, these are most likely due to the *in vitro* system applied, e.g. due to the limited genomic context used in the minigenes or tissue‐specific regulatory elements present in cone photoreceptors but lacking in HEK293T cells [16, 37]. Minigene assays are artificial systems that may not fully replicate the complex interactions and environment of and within cells natively expressing the gene of interest, possibly affecting the accuracy of the splicing analysis. To overcome these limitations, patient‐derived induced pluripotent stem cells can be differentiated in photoreceptor precursor cells [38], or even retinal organoids [39]. Yet such approaches are expensive and time‐consuming, and may not be implicated for a larger number of variants, as investigated in this study.

To demonstrate the suitability of minigene assays, we previously also analyzed splicing variants in patient‐derived samples. For a variant in *MFSD8* linked to neuronal ceroid lipofuscinosis and non‐syndromic retinopathy [40], as well as variants in *OPA1*‐associated with dominant optic atrophy [41], we found strong concordance between minigene assay results and patient‐derived cDNA analyses. Similar findings in other disease genes, such as *BRCA1*, further confirm this comparability [42].

The introduction of the selected variants into the minigene constructs allowed for a comparative analysis of splicing outcomes. We performed an in‐depth characterization of the RT‐PCR products by subcloning and sequencing in order to gain detailed information on the aberrant splicing profiles, as we consider this essential for correct variant assessment—a laborious strategy not always followed in studies evaluating candidate spliceogenic variants [43, 44, 45]. For the majority of variants (16/21, 76%), aberrant splicing patterns were observed (Table 1, supplementary material, Table S2), including exon skipping, intron retention, and pseudoexon inclusion, aligning with the *in silico* predictions. However, two variants (c.1178+9T>C and c.1578+12C>T) showed no aberrantly spliced transcripts, also confirming computational predictions.

For c.1178+6T>C, c.2103+4A>G, and the putative missense variant c.1778T>G;p.(Ile593Ser) the major observed transcript was correctly spliced. We do consider these variants as functionally abnormal with respect to the minigene assay, but the disease relevance of these variants remains uncertain. Therefore, we grade these variants as hypomorphic. A more clear‐cut outcome of cDNA analysis could potentially be achieved in photoreceptor‐like cells that express the aberrant transcript in a more native genomic context and with all necessary endogenous splicing factors. However, this was not the aim of this study.

The observed splicing abnormalities in most instances resulted in frame‐shift and PTC, suggesting that the resulting aberrant transcripts would likely be targeted for NMD (Table 1). The only exceptions were the variants c.2103G>C and c.2103+4A>G, which resulted in the use of different cryptic donor splice sites within intron 17. The resulting transcripts are not expected to undergo NMD. If translated, only the poorly conserved C‐terminus of CNGB3, located downstream of Gln701 or Lys702—and therefore downstream of the predicted CaM binding sites at amino acids 673–692—would be lost [9]. Whether this impairs the function of the CNG channel remains uncertain.

Only three of the aberrantly spliced transcripts identified resulted in in‐frame exon skipping. This was the case for Δ(Ex5), Δ(Ex8), and Δ(Ex10). These exons are naturally dividable by three (Table 1), resulting in the deletion of important amino acid sequences from TD1, TD3 to TD4, TD5, or the pore region, respectively (Table 1). Since this probably impairs protein folding of structurally important domains or protein flexibility, these in‐frame deletions are considered detrimental to channel function.

Six putative missense variants [c.643G>C;p.(Asp215His), c.989A>G;p.(Lys330Arg), c.1055G>A;p.(Arg352Lys), c.1778T>G;p.(Ile593Ser), c.1781G>C;p.(Ser594Thr), c.2103G>C;p.(Gln701His)] were included in the study. For all but variant c.1778T>G;p.(Ile593Ser), the minigene assay provided evidence that these variants result in aberrant splicing and likely represent spliceogenic rather than missense variants. For variant c.1778T>G;p.(Ile593Ser) further functional *in vitro* analysis is required using our aequorin‐based bioassay, similar to our previous study on the functional characterization of variants in *CNGA3* [5]. This analysis is currently underway and may help to further elucidate the pathogenic effects of this variant.

For *CNGB3*, three deep intronic variants have been previously identified and functionally evaluated [11, 46]. In this study, two new deep intronic spliceogenic variants (c.903+1711G>T and c.1578+2509G>C) have been added to this important variant class in *CNGB3*.

The functional evidence obtained from the minigene assays significantly influenced the classification of variants according to the ACMG/AMP guidelines [19, 20]. Twelve of fourteen (86%) variants initially categorized as VUS were upgraded to likely pathogenic or pathogenic, enhancing the clinical utility of the genetic data. This reclassification underscores the importance of functional assays in supplementing *in silico* predictions, providing a more accurate assessment of variant pathogenicity.

In conclusion, this study demonstrates the importance, utility, and limitations of minigene assays in evaluating splicing defects induced by single‐nucleotide variants in the *CNGB3* gene. While *in silico* tools provide valuable initial insights, experimental validation remains crucial for accurate variant classification, ultimately aiding in the diagnosis and management of ACHM and IRD.

## Author contributions statement

PR, NW and SK conceived the experiments. KR and PR conducted the experiments and analyzed the data. All authors were involved in writing the article and had final approval of the submitted and published versions.

## Supporting information


**Figure S1.** Electropherograms of the *CNGB3* wildtype minigene splice products
**Figure S2.** *CNGB3* RNA‐seq data from human post‐mortem macular retina (NCBI BioProject PRJNA369687)
**Figure S3.** Electropherograms of the mutant *CNGB3* minigene splice products
**Figure S4.** Comparison and quantification of observed splice products for minigene Ex7–8
**Figure S5.** Comparison and quantification of observed minigene splice products for minigene Ex15
**Figure S6.** Comparison and quantification of observed minigene splice products for minigene Ex16–17

**Table S1.** Primer used for cloning of the *CNGB3* minigenes
**Table S2.** Variant location on transcript, protein and genomic level, source of the variant, variant frequency in the normal population as well as ACMG/AMP classification and criteria before and after application of the functional minigene data are provided
**Table S3.** Unpublished variants (bold) presented in this study alongside the genotype observed in the respective ACHM patient
**Table S4.** Splice products observed for wildtype minigenes

## Data Availability

The data that support the findings of this study are available from the corresponding author upon reasonable request.
