# Supplementary material for: Comprehensive functional splicing analysis of non‐canonical CNGB3 variants using in vitro minigene splice assays
Source: J Pathol. 2025 Apr 30;266(3):322–36. doi: 10.1002/path.6431 (PMC12146803; doi:10.1002/path.6431)
Supplement: Supplementary file 1 — Figure S1. Electropherograms of the CNGB3 wildtype minigene splice products Figure S2. CNGB3 RNA‐seq data from human post‐mortem macular retina (NCBI BioProject PRJNA369687) Figure S3. Electropherograms of the mutant CNGB3 minigene splice products Figure S4. Comparison and quantification of observed splice products for minigene Ex7–8 Figure S5. Comparison and quantification of observed minigene splice products for minigene Ex15 Figure S6. Comparison and quantification of observed minigene splice products for minigene Ex16–17 [file PATH-266-322-s002.docx]

**Comprehensive functional splicing analysis of noncanonical *CNGB3* variants using *in vitro* minigene splice assays**

K Rawnsley *et al.* *J Pathol* <https://doi.org/10.1002/path.6431>

**Supplementary Figures S1–S6**

**Supplementary Tables S1–S4 (provided in a separate Excel file)**

Reference numbers refer to the main text list.


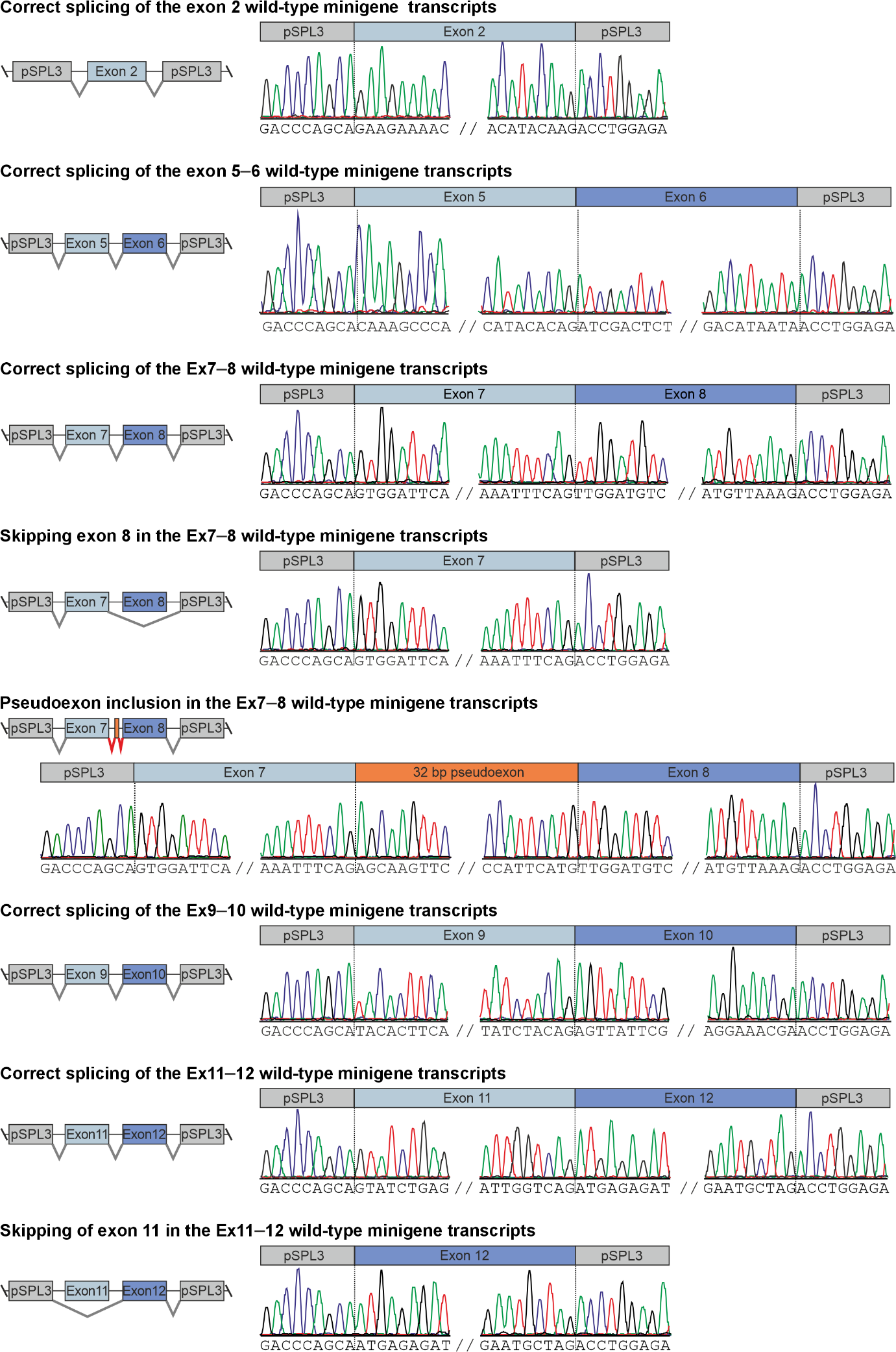


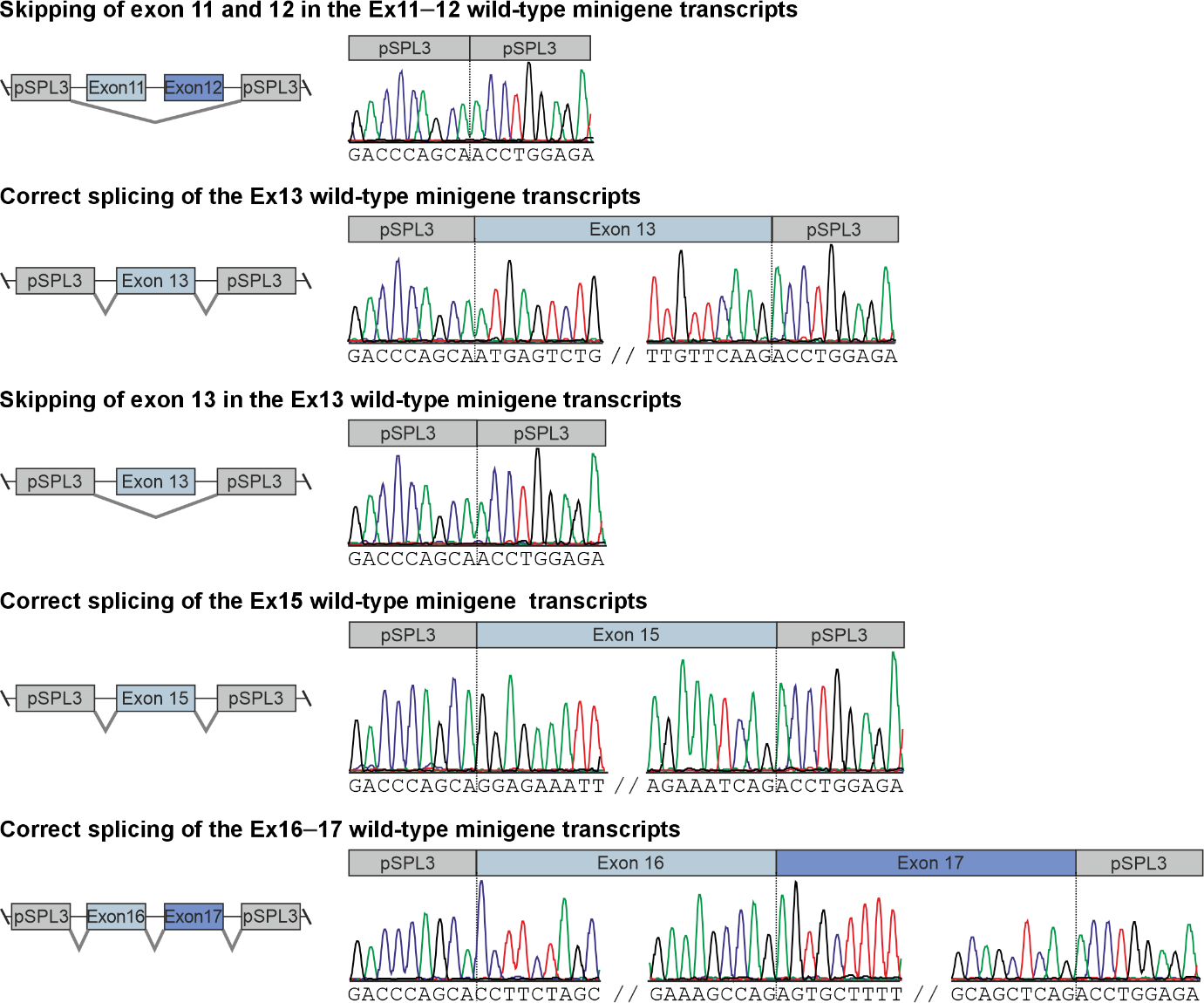


**Figure S1. Electropherograms of the *CNGB3* wildtype minigene splice products.** Left: Schematic composition of the nine different minigenes with one or two *CNGB3* exons located between the pSPL3-specific vector exons. Gray triangles indicate the observed splicing events. Right: Electropherograms of the wildtype minigene splice products after cDNA analysis documenting the splice junctions. For all nine minigenes, the expected wildtype splicing event was observed. Three minigenes presented with additional splice products: For minigenes Ex7–8, skipping of exon 8 and a 32-bp pseudoexon inclusion was observed. For minigene Ex11–12, skipping of exon 11 or exon 11 and exon 12 were detected. Exon 13 skipping has also been demonstrated for the Ex13 minigene.

**
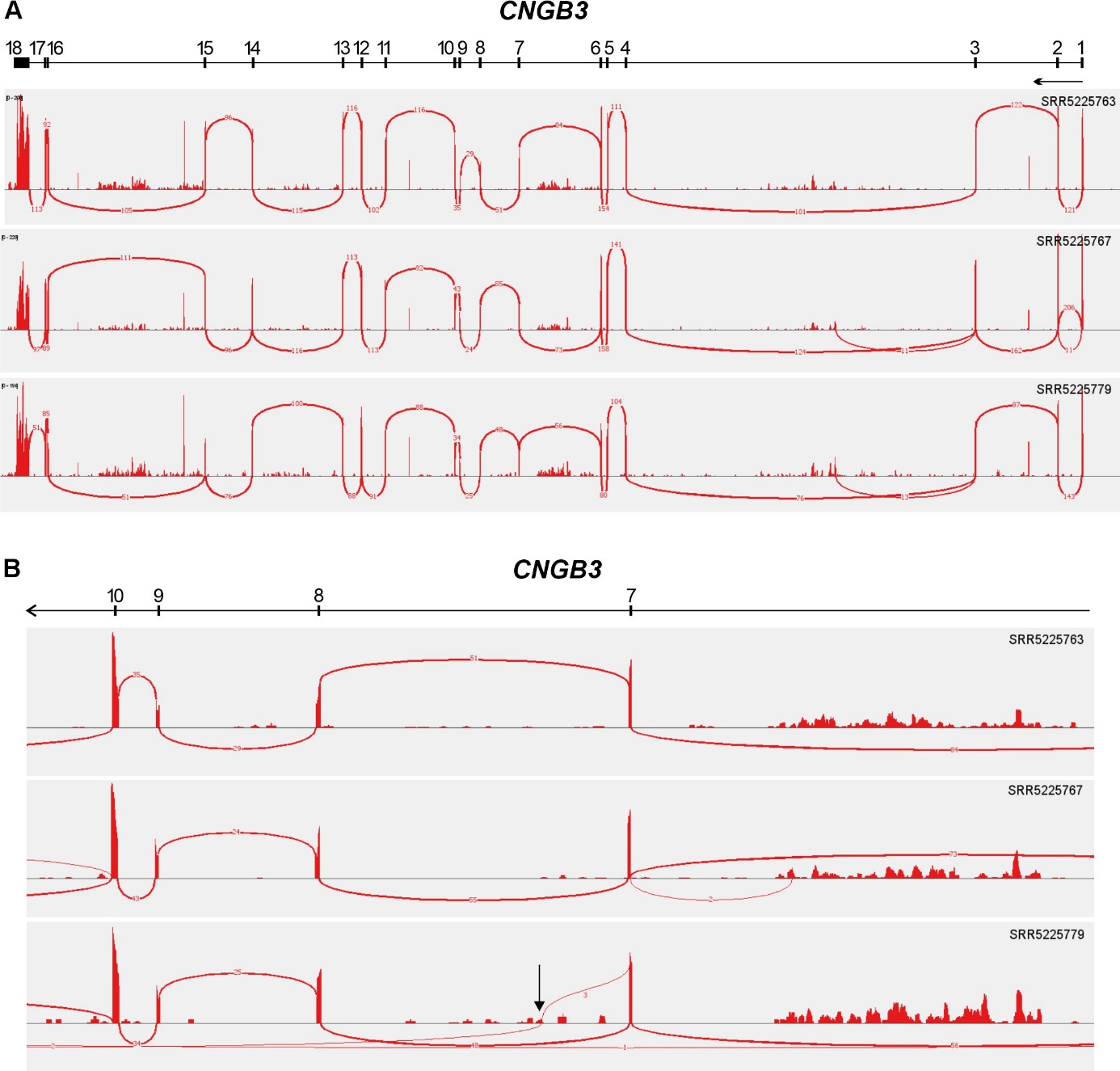
**

**Figure S2. *CNGB3* RNA-seq data from human *postmortem* macular retina (NCBI BioProject PRJNA369687).** (A) Shashimi plots of three different samples show that *CNGB3* comprises 18 exons, all of which are constitutively spliced. Exon (black boxes) and intron (black line) structure is depicted in the upper panel with the arrow indicating orientation of the *CNGB3* gene on the plus strand. Minimum splice site coverage was set to 10. (B) In one (SRR5225779) of the three samples, recognition of a 32-bp pseudoexon (black arrow) within intron 7 is observed in the Shashimi plots. Minimum splice site coverage was defined as 1.

**
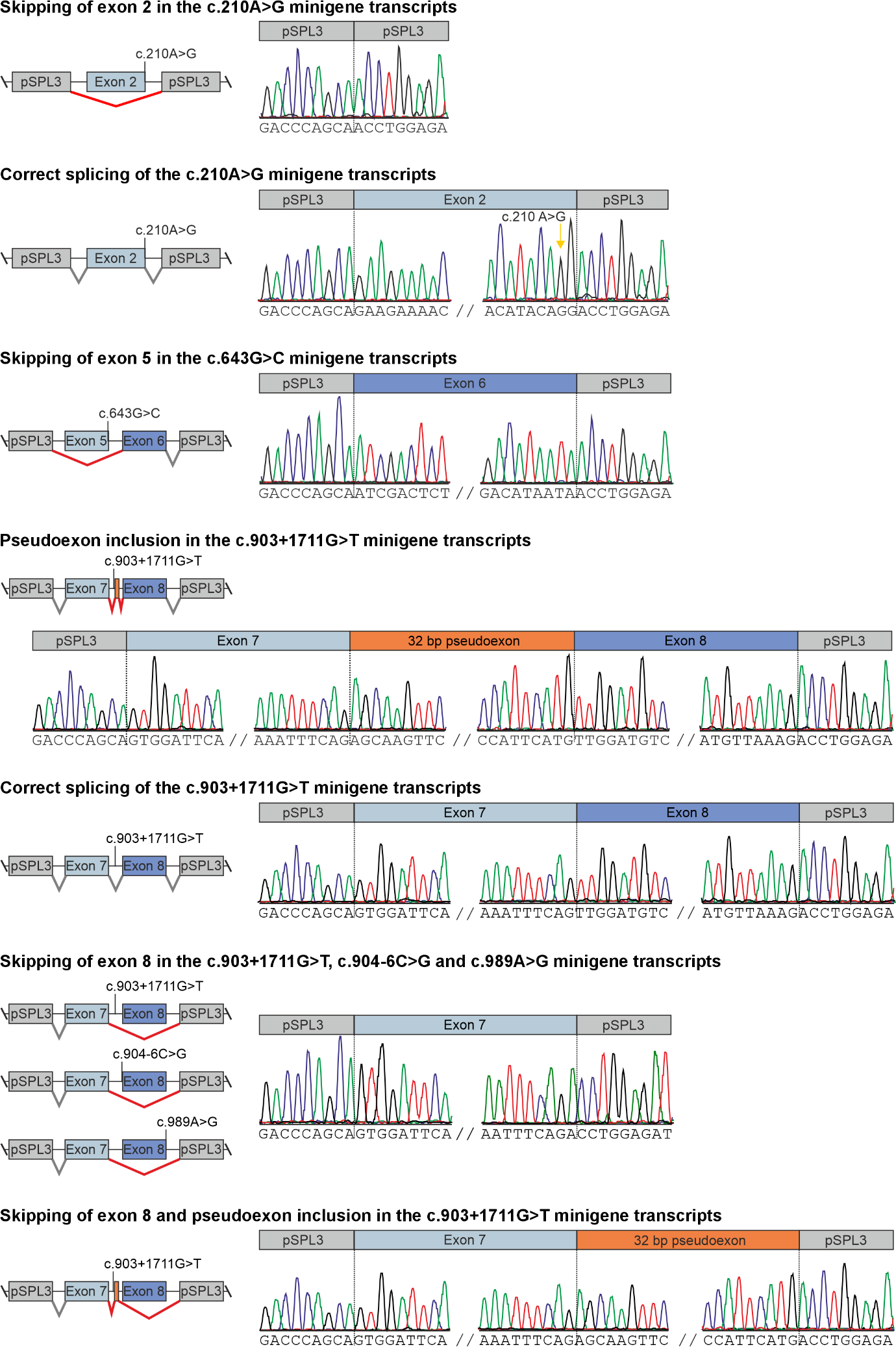
**

**
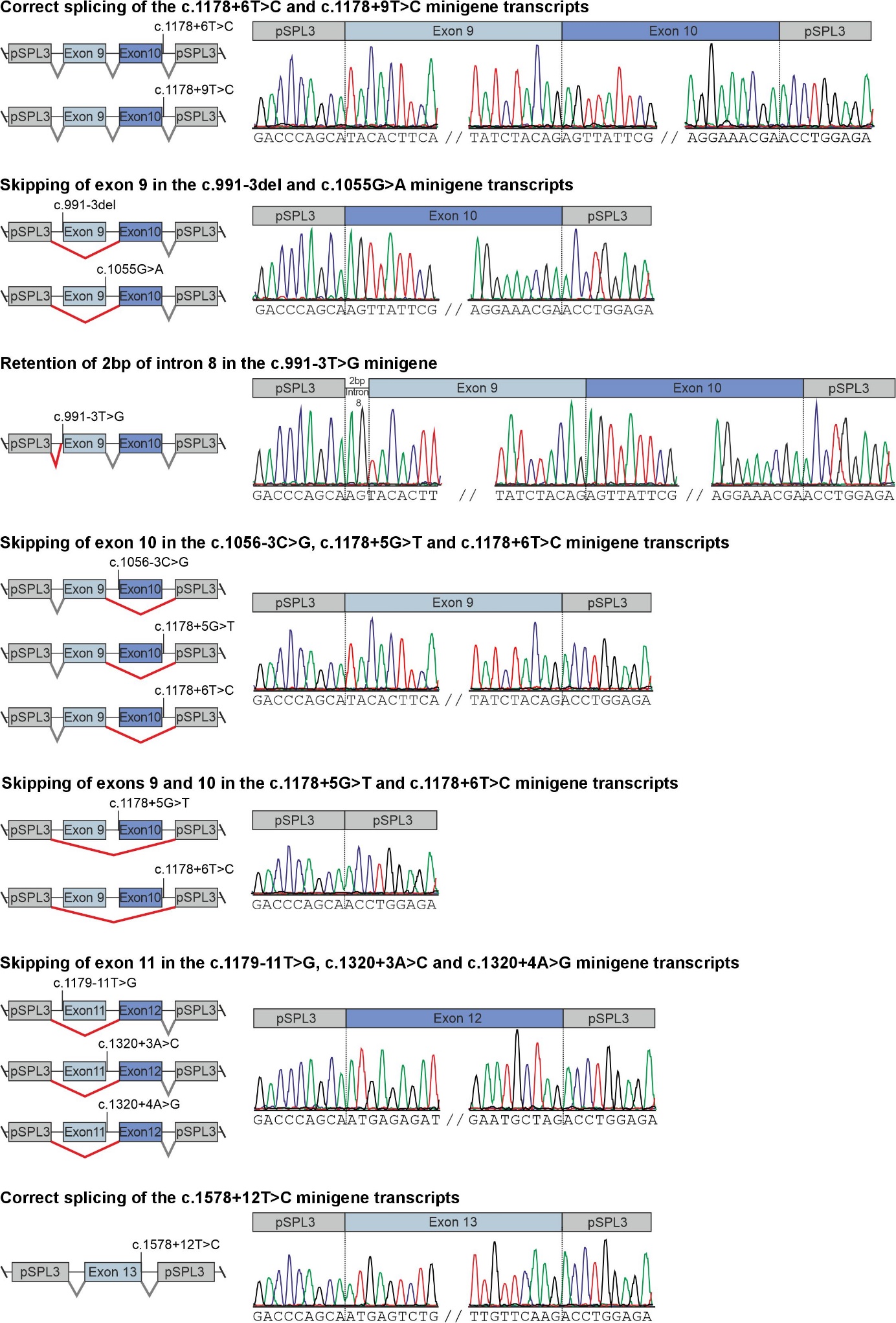

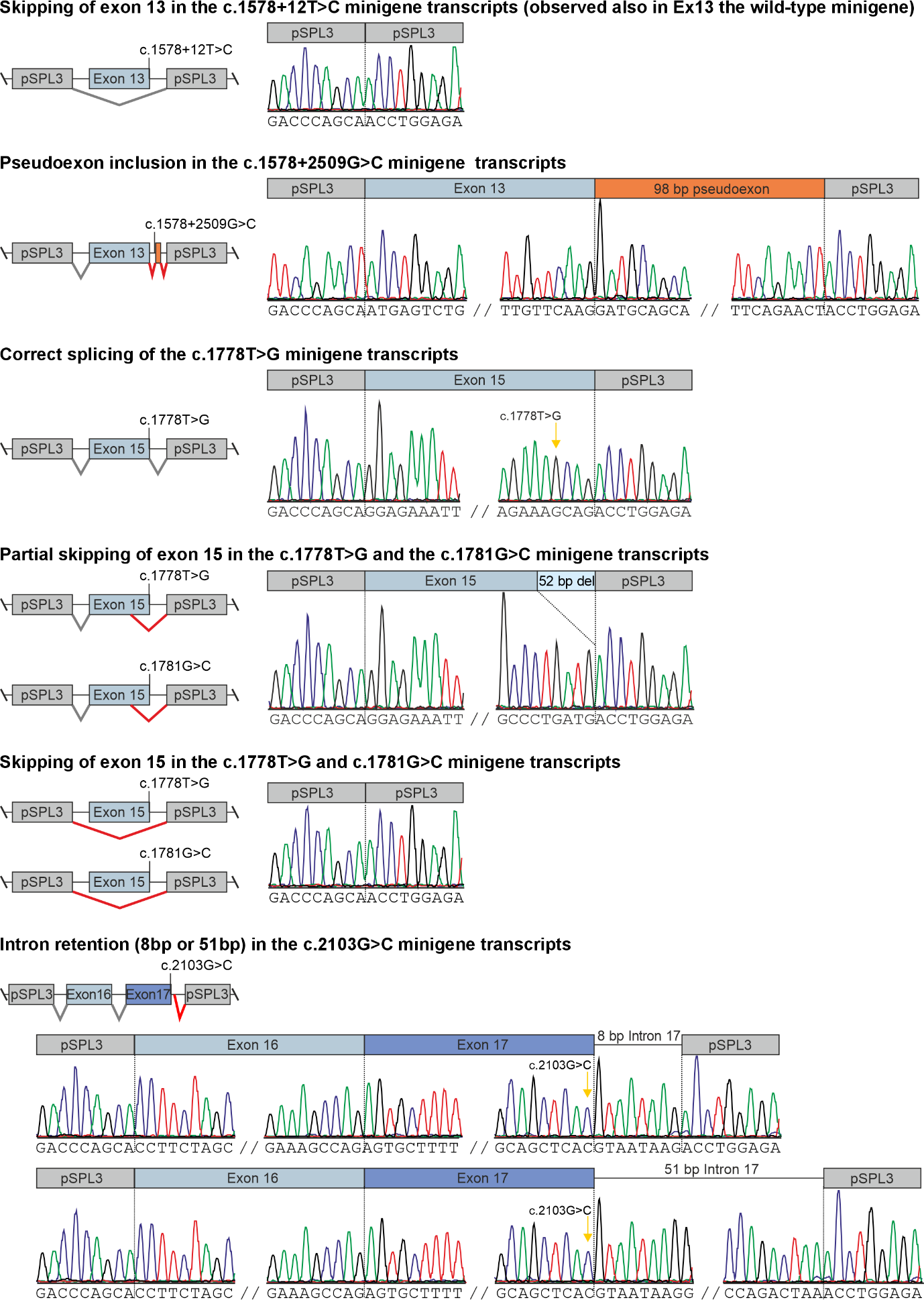

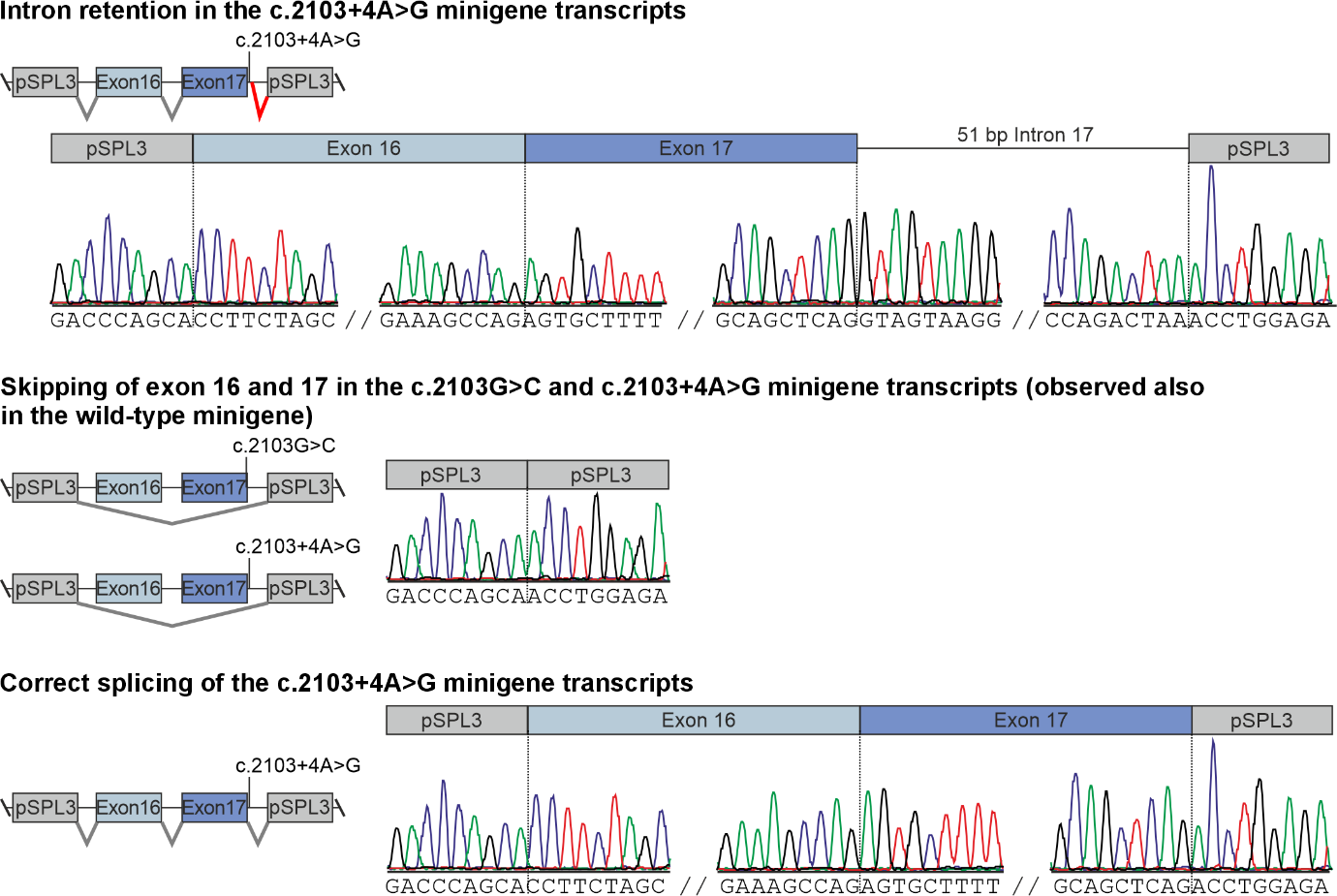
**

**Figure S3. Electropherograms of the mutant *CNGB3* minigene splice products.** Left: Schematic composition of the nine different minigenes with one or two exons intervening the pSPL3-specific vector exons and location of the single variants tested. Gray triangles indicate correct splicing, whereas red triangles denote aberrant splicing events. Right: Electropherograms of the mutant minigene splice products after cDNA analysis documenting the splice junctions.

**
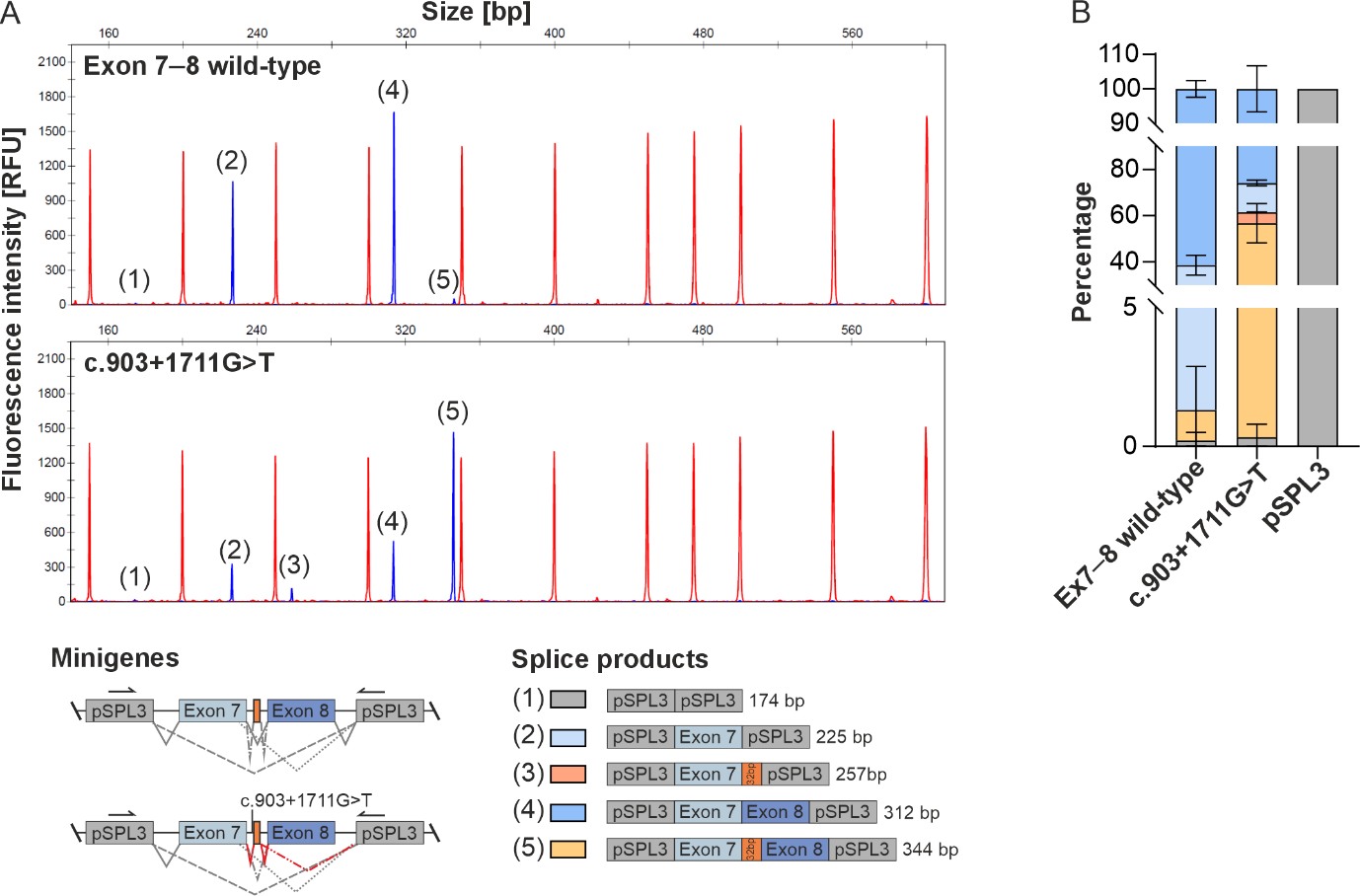
**

**Figure S4. Comparison and quantification of observed splice products for minigene Ex7–8.** (A) Top: Capillary fragment analysis for the wildtype minigene splice products and Ex7–8 minigene carrying the variant c.903+1711G>T. The size marker is given in red, while the tested fragments are visualized in blue. Below: Schematic presentation of the observed splicing events (left) and the resultant splice products (right). (B) Relative quantification of the different minigene splice products resulting from the wildtype and mutant minigenes and the empty pSPL3 vector used as a control. RFU: relative fluorescence unit.

**
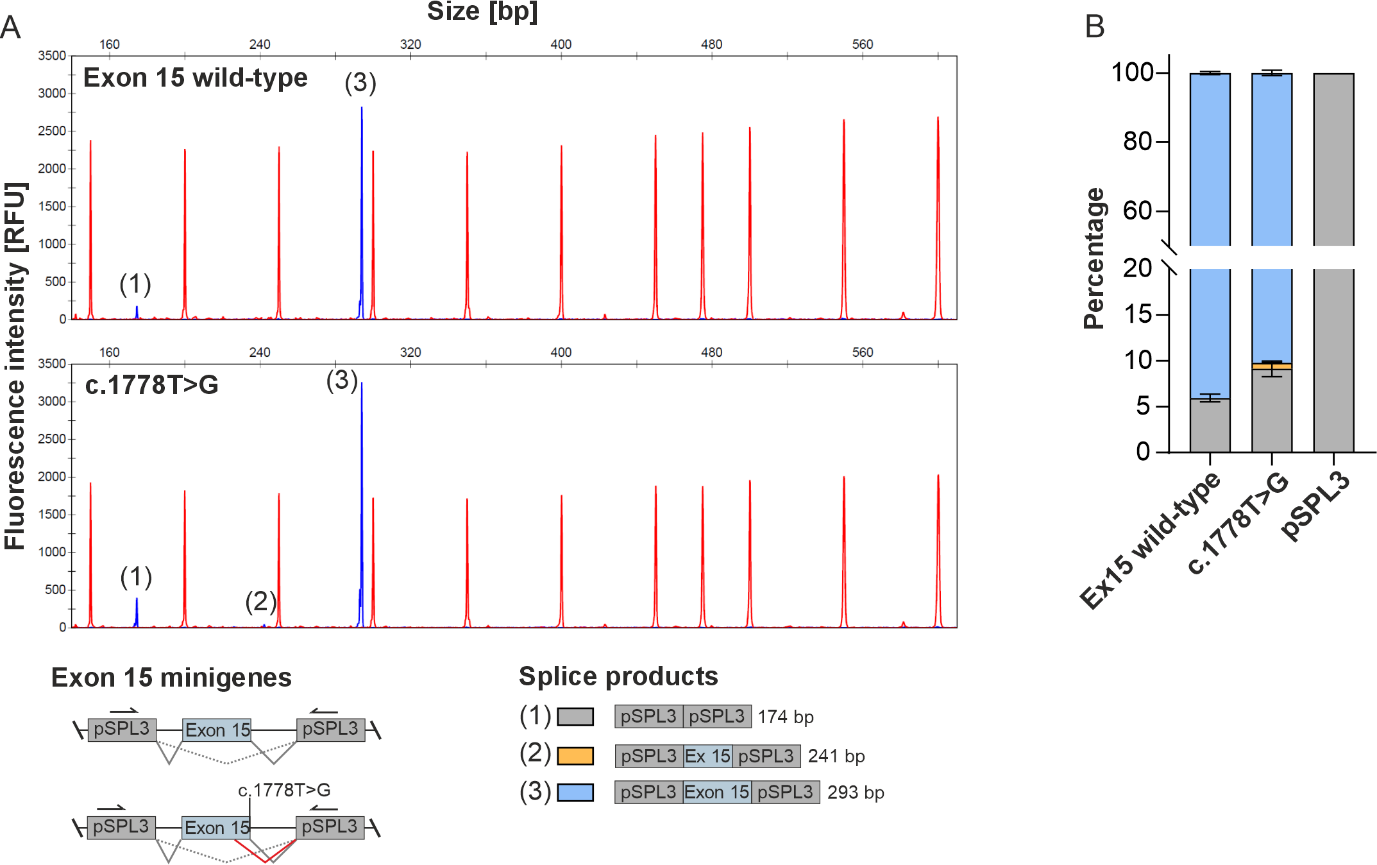
**

**Figure S5. Comparison and quantification of observed minigene splice products for minigene Ex15.** (A) Top: Capillary fragment analysis for the wildtype minigene splice products and Ex15 minigene carrying the variant c.1778T>G. The size marker is given in red, while the tested fragments are visualized in blue. Below: Schematic presentation of the observed splicing events (left) and the resultant splice products (right). (B) Relative quantification of the different minigene splice products resulting from the wildtype and mutant minigenes and the empty pSPL3 vector used as a control. RFU: relative fluorescence unit.

**
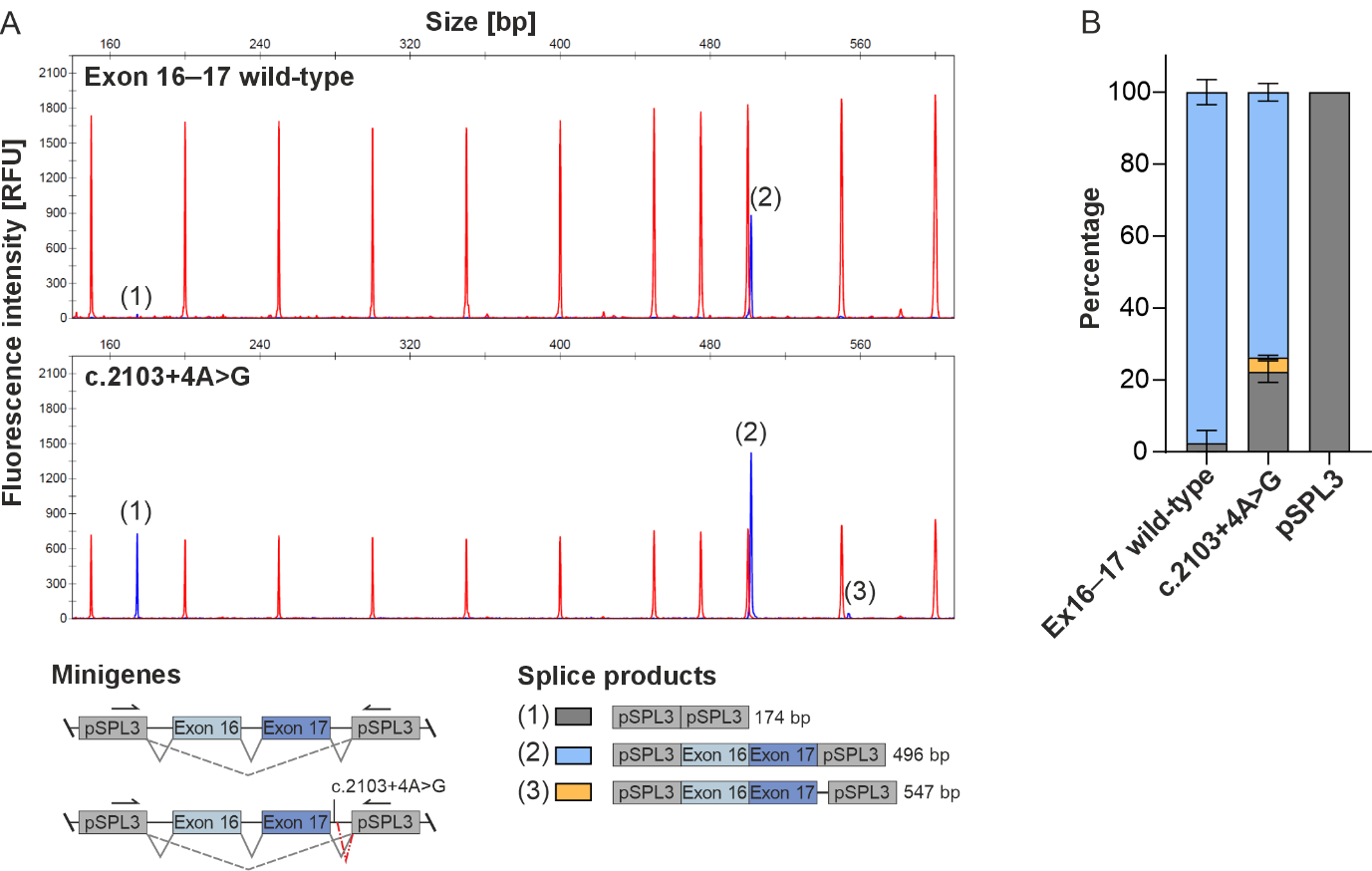
**

**Figure S6. Comparison and quantification of observed minigene splice products for minigene Ex16–17.** (A) Top: Capillary fragment analysis for the wildtype minigene splice products and Ex16–17 minigene carrying the variant c.1778T>G. The size marker is given in red, while the tested fragments are visualized in blue. Below: Schematic presentation of the observed splicing events (left) and the resultant splice products (right). (B) Relative quantification of the different minigene splice products resulting from the wildtype and mutant minigenes and the empty pSPL3 vector used as a control. RFU: relative fluorescence unit.
